# Oxygen-Content-Dependent Interfacial and Barrier Effects of Graphene Fillers in PVA Adhesives Toward Durable Polarizer Applications

**DOI:** 10.3390/polym18151916

**Published:** 2026-08-05

**Authors:** Chang Sun, Wentao Huang, Ziyuan Zheng, Rui Huang, Qinghua Zhao, Guohua Chen

**Affiliations:** 1 College of Materials Science and Engineering, Huaqiao University, Xiamen 361021, China; 2 Xiamen Xiangfuxing Technology Co., Ltd., Xiamen 361021, China

**Keywords:** waterborne PVA adhesive, graphene derivatives, oxygen content, water vapor barrier, interfacial interaction

## Abstract

Waterborne poly(vinyl alcohol) (PVA) adhesives are widely used in iodine-based polarizers, owing to their excellent transparency and interfacial adhesion. However, the intrinsic hydrophilicity of PVA compromises the long-term durability of polarizers under humid conditions. Herein, graphene derivatives with tunable oxygen contents and graphitization degrees, including graphene oxide (GO), partially reduced graphene oxide (rGO), and graphene nanosheets (GNs), were incorporated into a PVA/PEI adhesive system to investigate the oxygen-content-dependent interfacial interactions and moisture-barrier mechanisms. Structural analyses reveal that oxygen-rich GO enhances interfacial hydrogen bonding and polymer–graphene interactions, whereas highly graphitized GN primarily functions through its intrinsic lamellar barrier effect by increasing diffusion tortuosity and reducing water affinity. The rGO exhibits a compromise between interfacial interactions and barrier effects due to its moderate oxygen content and preserved graphene structure. Among them, the GN-modified adhesive demonstrates the most favorable overall performances, achieving a 14.71% reduction in the water vapor transmission rate (WVTR) of the assembled polarizer, enhanced moisture resistance, and improved antistatic capability while maintaining acceptable optical transparency. Furthermore, practical polarizer evaluations confirm that GN effectively suppresses moisture penetration, with only slight bubbling observed after 8 days of water immersion and no delamination or polarization degradation during a 40-day immersion test. These findings provide insights into the relationship between graphene oxygen content, interfacial interactions, and moisture-barrier behavior, offering an effective strategy for designing durable multifunctional waterborne adhesives for advanced optoelectronic polarizer applications.

## 1. Introduction

Iodine-doped poly(vinyl alcohol) (PVA) polarizers are widely used in modern liquid-crystal displays (LCDs), owing to their high optical transmittance, excellent polarization efficiency, and low chromatic distortion [1,2]. However, the intrinsic hydrophilicity of PVA makes iodine-based polarizers highly susceptible to moisture-induced degradation. Water penetration can cause swelling of the PVA matrix, destabilization of polyiodide complexes, and deterioration of interfacial adhesion, ultimately leading to optical attenuation, bubbling, and delamination during long-term operation in humid conditions [3,4]. Therefore, improving the moisture resistance and environmental durability of PVA-based polarizers remains a critical challenge for advanced display technologies [5].

A typical polarizer consists of an iodine-doped PVA layer laminated between two triacetyl cellulose (TAC) protective films using waterborne PVA adhesives. Although PVA adhesives provide excellent transparency and adhesion, their abundant hydroxyl groups facilitate water absorption and vapor diffusion, resulting in poor moisture-barrier performance. Conventional crosslinking strategies can partially improve water resistance [6,7,8,9,10,11,12], but often sacrifice optical transparency, flexibility, or processability. Consequently, developing multifunctional PVA adhesives that simultaneously exhibit strong interfacial adhesion, effective moisture-barrier properties, and long-term optical stability is highly desirable.

Graphene derivatives have attracted extensive attention as barrier nanofillers because of their exceptional impermeability and high aspect ratios [13,14,15]. Defect-free graphene sheets have been demonstrated to be nearly impermeable to gases, while layered graphene structures can effectively prolong molecular diffusion pathways through a tortuous-path effect [16,17]. In addition, oxygen-containing functional groups on graphene surfaces can participate in hydrogen bonding and polymer–graphene interactions, thereby enhancing interfacial adhesion and network integrity [18,19]. Depending on the oxygen content and graphitization degree, graphene oxide (GO), reduced graphene oxide (rGO), and graphene nanosheets (GNs) exhibit distinct interfacial and barrier characteristics [20,21,22,23]. GO possesses abundant oxygen-containing functional groups that promote strong interfacial interactions, whereas highly graphitized GN provides superior intrinsic moisture-barrier performance through its lamellar structure. rGO represents an intermediate structure balancing these two effects. Benefiting from outstanding optical features, graphene materials also show promising prospects in display and optoelectronic devices [24]. Despite extensive studies on graphene/polymer composites, the role of graphene oxygen content in regulating the balance between interfacial interactions and intrinsic barrier effects remains insufficiently understood, particularly in waterborne PVA adhesive systems for iodine-based polarizers.

In this study, graphene derivatives with progressively decreased oxygen contents, including GO, rGO, and GN, were incorporated into a PVA/PEI adhesive matrix to elucidate the oxygen-content-dependent interfacial and barrier mechanisms governing moisture resistance. The relationships among graphene surface chemistry, interfacial interactions, moisture diffusion behavior, and polarizer durability were systematically investigated. This work provides new insights into the rational design of high-performance waterborne adhesives for durable optoelectronic polarizer applications. Detailed performance trends of various filler loadings and rGO reduction durations are provided in the Supplementary Information for reference.

## 2. Materials and Methods

### 2.1. Materials

Sulfuric acid (H_2_SO_4_, ≥99%), potassium permanganate (KMnO_4_, 99%), hydrogen peroxide (H_2_O_2_, 30 wt%), sodium hydroxide (NaOH), and L-ascorbic acid (L-AA) were purchased from Sinopharm Chemical Reagent Co., Ltd., Beijing, China. Polyvinyl alcohol (PVA), polyethyleneimine (PEI), and zirconyl nitrate (ZrO(NO_3_)_2_, 99%) were used as received. Flake graphite (8000 mesh), graphene microflakes, and PVA-polarizing films were supplied by Xiamen Kaina Graphene Technology Co., Ltd., Xiamen, China. Triacetyl cellulose (TAC) films were provided by Dahui Optoelectronics Co., Ltd., Xiamen, China.

### 2.2. Preparation of GO

Graphene oxide was prepared using a modified Hummers method. The simple synthetic method was as follows. First, 200 mL of sulfuric acid (H_2_SO_4_) and 4 g of graphite flakes (8000 M) were mixed in an ice bath at 0 °C. After the solution was mixed and dispersed, 24 g of potassium permanganate (KMnO_4_) was added slowly several times, and the solution was stirred for 30 min. After an ice bath, the mixed solution was transferred to a constant-temperature water bath at 35 °C, and continued stirring for 2 h. Then, a high-temperature reaction was carried out by slowly adding ultra-pure water to keep the temperature at 90 °C–110 °C, until the temperature no longer increased after the addition of water. Finally, after cooling, 30% hydrogen peroxide was added drop by drop and the solution turned golden yellow. This solution was washed several times by ultra-pure water, neutralized to PH = 6–7, and freeze-dried to obtain GO.

### 2.3. Preparation of Modified Adhesives

#### 2.3.1. Preparation of GO Adhesives

The PVA solution was first prepared. Ultra-pure water and PVA were mixed in a 30 °C water bath and dissolved with stirring for 30 min. Then, the mixture was heated in a water bath at 95 °C for 2 h and cooled naturally to room temperature to obtain a PVA solution with a solid content of 5 wt%. The GO was weighed and added to the PVA (5 wt%) in a certain proportion and stirred for 30 min. Then, 3 wt% PEI (4%) was slowly added drop by drop into the solution, using a burette with stirring for 30 min. Subsequently, 6 wt% of ZrO(NO_3_)_2_ (10%) was weighed and added to the solution slowly drop by drop using a burette with stirring for 30 min, and the GO adhesives were obtained by filtering out air bubbles through a strainer after standing.

#### 2.3.2. Preparation of rGO Adhesives

The rGO adhesives were prepared via a post-reduction strategy following the formation of the GO-crosslinked adhesive network. Briefly, a GO dispersion (0.08 wt%) was first added to a 5 wt% PVA solution and stirred for 30 min to obtain a homogeneous mixture. Subsequently, a 3 wt% PEI solution (4 wt%) was slowly introduced dropwise under continuous stirring for 30 min, followed by the addition of a 6 wt% ZrO(NO_3_)_2_ solution (10 wt%) under stirring for another 30 min to construct the crosslinked GO/PVA adhesive network. After crosslinking, L-ascorbic acid (L-AA) was added as a reducing agent at a mass ratio of GO:L-AA = 1:6. The reduction process was carried out in a 60 °C water bath for different durations to obtain rGO-AA adhesives with tunable reduction degrees. No additional washing or purification was performed after the reduction process to preserve the integrity of the crosslinked adhesive network, and the adhesive was directly used in its as-prepared state. Finally, the resulting adhesive was degassed and filtered to remove air bubbles before further use.

#### 2.3.3. Preparation of GN Adhesives

The graphene microflakes (G2) possessed a median lateral size (D50) of approximately 8.9 μm, with an average thickness of 2.57 nm (approximately 9–10 layers). G2 was used as the graphene precursor for preparing the GN, and its intrinsic chemical structure was characterized before the ball-milling treatment. Due to the relatively large thickness and multilayer structure of G2, a planetary ball-milling process was employed to promote partial exfoliation and improve its dispersion in the PVA matrix. The 5 wt% PVA solution and graphene microflakes (G2) were mixed at a solid-content ratio of 1:0.0005 and transferred into an agate ball-milling jar. The mass ratio of the mixed solution to the agate milling beads was 1:2. The jar was mounted on a planetary ball mill and milled at 40 Hz for 24 h to obtain a 0.05 wt% GN/PVA dispersion. The obtained GN/PVA dispersion (0.05 wt%) was added to the 5 wt% PVA solution at the desired proportion and stirred for 30 min. Then, a 3 wt% PEI solution (4 wt%) was added dropwise using a burette under continuous stirring for 30 min. Subsequently, a 6 wt% ZrO(NO_3_)_2_ solution (10 wt%) was added dropwise using a burette under continuous stirring for another 30 min. Finally, the adhesive was allowed to stand for degassing and then filtered before use.

### 2.4. Preparation of Modified Adhesive Films

The GO adhesive was poured into the mold, put into the vacuum-drying oven, the temperature was adjusted to 60 °C, and it was dried for 24 h. GO adhesive films with a thickness of 70 μm were prepared. rGO adhesive films and GN adhesive films were prepared in the same way as above.

### 2.5. Preparation of Modified Polarized Base Films

The configured aqueous adhesive was passed through the production line of Xiangfu Xing Technology Company (Xiamen, China) Limited for the attachment of polarized base film (Figure S1). The two layers of saponified TAC film were laminated with the PVA-polarizing film, which was prepared by wet stretching, using different adhesives and adhesive-roll laminating equipment to obtain the polarized base film (POL-B), GO polarized base film (GO-POL-B), rGO polarized base film (rGO-POL,-B) and GN polarized base film (GN-POL-B).

### 2.6. Characterization

Crystallinity tests were performed using an X-ray diffractometer (D8-Advance, Bruker, Karlsruhe, Germany), with a radiation tube voltage and current of 40 kV and 40 mA, a scanning range from 5° to 50°, and a scanning speed of 5°/min. The chemical structures of the prepared films were observed using the ATR mode of a Nicolet iS 50 FTIR spectrometer (Thermo Fisher Scientific, Waltham, MA, USA). All spectra were scanned within the mid-infrared optical region (4000 cm^−1^ to 400 cm^−1^) with a resolution of 0.4 cm^−1^. The film embrittlement surfaces (liquid nitrogen embrittlement) were observed using field emission scanning electron microscopy (S-4800(II), Hitachi, Tokyo, Japan) with an operating voltage of 5 kV, an operating current of 10 mA and an accelerating voltage of 3 kV. The microstructures and ultrastructures of the samples were observed using transmission electron microscopy (JEM-2100, JEOL, Tokyo, Japan) at a field emission voltage of 200 kV. The film samples were clamped onto the molds and the UV transmittance of the samples was tested using a UV–visible spectrophotometer (Evolution201, Thermo Fisher Scientific, Waltham, MA, USA) in the range of 700 nm to 200 nm. The water vapor barrier of the sample was characterized using a water vapor transmission rate tester (W3/010, Jinan Labthink Instruments Co., Ltd., Jinan, China). The test conditions were 38 °C and 90% relative humidity and the test time was adjusted according to the water vapor transmission rate of the film samples. The surface resistivity of the adhesive films was measured using a surface resistance tester (740 SRM, FRASER Anti-Static Technology, London, UK) equipped with a two-electrode configuration. The measurements were performed at room temperature under ambient conditions, and the results were recorded in Ω/sq. The obtained surface resistivity values were used to evaluate the charge dissipation capability of the adhesive films. In accordance with GB/T 30693-2014 “Measurement of the contact angle between plastic film and water” [25], a contact-angle measuring instrument (JC2000D6, Shanghai Zhongchen Digital Equipment Co., Ltd., Shanghai, China) was used and the contact-angle value was fitted by the five-point fitting method.

The samples (30 mm × 30 mm) were weighed (*m*_0_, g) after being placed in an oven at 50 °C and dried to a constant weight. Subsequently immersed in ultra-pure water (1 min, 3 min, 5 min, 1 h, and 24 h), they were removed and weighed by blotting the excess water from the surface with dust-free paper (*m*_1_, g). They were then placed in an oven at 50 °C to dry to constant weight and weighed (*m*_2_, g). The water absorption and solubility of the samples can be calculated using Equations (1) and (2):

(1) $$A=\frac{m_{1}-m_{0}}{m_{0}}\times 100\%$$

(2) $$S=\frac{m_{0}-m_{2}}{m_{0}}\times 100\%$$

where *A* is water absorption (%) and *S* is solubility (%).

## 3. Results

### 3.1. Oxygen-Content-Dependent Interfacial Crosslinking and PVA Crystallinity

**Figure 1:**
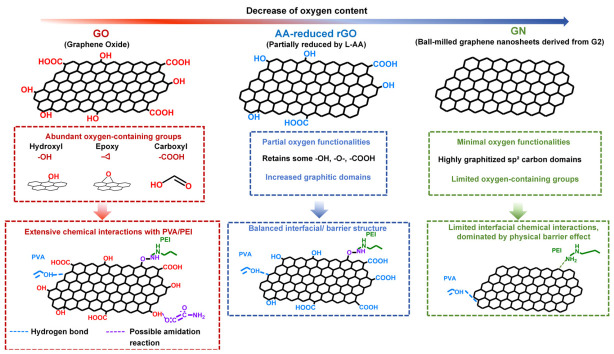
Schematic illustration of the oxygen-content-dependent structural characteristics of GO, AA-reduced rGO, and graphene nanosheets (GNs), and their proposed interactions with the PVA/PEI adhesive matrix.

The interfacial interactions between graphene-based fillers and the PVA adhesive matrix are primarily governed by the surface oxygen functionalities and graphitic structures of graphene. To elucidate the influence of oxygen content on interfacial interactions and moisture-resistant network formation, three graphene fillers with different oxygen levels, including graphene oxide (GO), AA-reduced graphene oxide (rGO), and ball-milled graphene nanosheets (GNs), were incorporated into the PVA/PEI/ZrO(NO_3_)_2_ adhesive system. As illustrated in Figure 1, GO contains abundant oxygen-containing functional groups, including hydroxyl, epoxy, and carboxyl groups, which facilitate strong hydrogen-bonding interactions with PVA/PEI chains and may promote additional interfacial interactions within the adhesive network [26]. In contrast, GN derived from commercial graphene microflakes (G2) possesses highly graphitized sp^2^ carbon domains and minimal oxygen functionalities, resulting in limited chemical interactions with the polymer matrix but enhanced physical barrier effects through its intrinsic lamellar structure. The AA-reduced rGO retains a certain amount of oxygen-containing groups while recovering partially graphitic domains, thereby providing a balance between interfacial compatibility and barrier performance.

**Figure 2:**
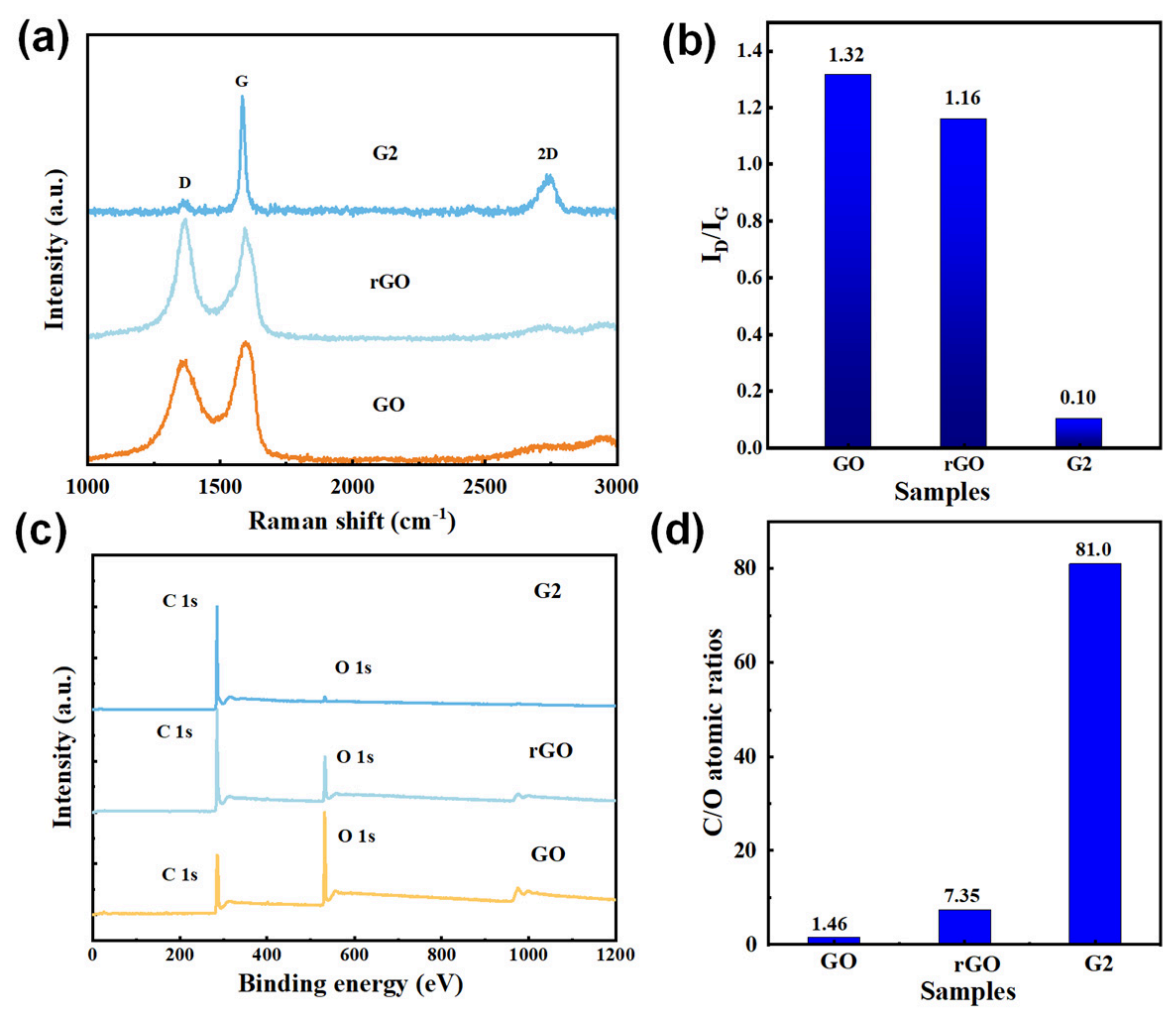
Oxygen content and structural evolution of graphene-based fillers. (**a**) Raman spectra and (**b**) corresponding ID/IG ratios of GO, rGO, and G2 graphene microflakes. (**c**) XPS survey spectra and (**d**) C/O atomic ratios calculated from XPS elemental compositions.

To verify the structural and chemical differences among the selected graphene-based fillers, Raman spectroscopy and XPS analyses were performed. As shown in Figure 2a, all samples exhibit the characteristic D and G bands of graphene-based materials. The I_D_/I_G_ ratios of GO, rGO, and G2 are 1.32, 1.16, and 0.10, respectively (Figure 2b). Compared with GO, the decreased I_D_/I_G_ ratio of rGO after L-ascorbic acid reduction indicates partial restoration of the sp^2^ carbon network and increased graphitic ordering. In comparison, G2 exhibits a much lower I_D_/I_G_ ratio, confirming its highly graphitized carbon structure with fewer lattice defects. The chemical compositions and oxygen contents of the graphene-based fillers were further investigated by XPS. The survey spectra (Figure 2c) display characteristic C 1s and O 1s signals, while the relative intensity of the oxygen-related signal decreases significantly from GO to rGO and G2. The calculated C/O atomic ratios increase from 1.46 for GO to 7.35 for rGO and 81.0 for G2 (Figure 2d), demonstrating the distinct oxygen levels and surface chemical characteristics among the fillers. The high-resolution C 1s and O 1s spectra (Figure S2) further reveal the evolution of oxygen-containing carbon species, and the detailed elemental compositions and C/O atomic ratios are summarized in Table S1. The decreased contributions of oxygen-containing carbon components after reduction and the weakened O 1s signals further confirm the removal of surface oxygen functionalities. These results demonstrate that GO, rGO, and G2 possess distinctly different surface chemistries and graphitic structures, providing the structural basis for understanding their different interfacial behaviors within the PVA adhesive matrix.

Based on the distinct oxygen contents and structural characteristics confirmed above, three representative graphene fillers, namely GO (0.08 wt%), rGO (24 h reduction), and GN (0.04 wt%), were selected for further investigation of their interfacial interactions within the PVA adhesive matrix. Each selected loading or reduction condition exhibited the optimal overall performance within its corresponding series. These concentrations were determined based on dispersion stability and film-forming behavior, while the complete FT-IR and XRD results under different conditions are provided in Figures S3 and S4.

**Figure 3:**
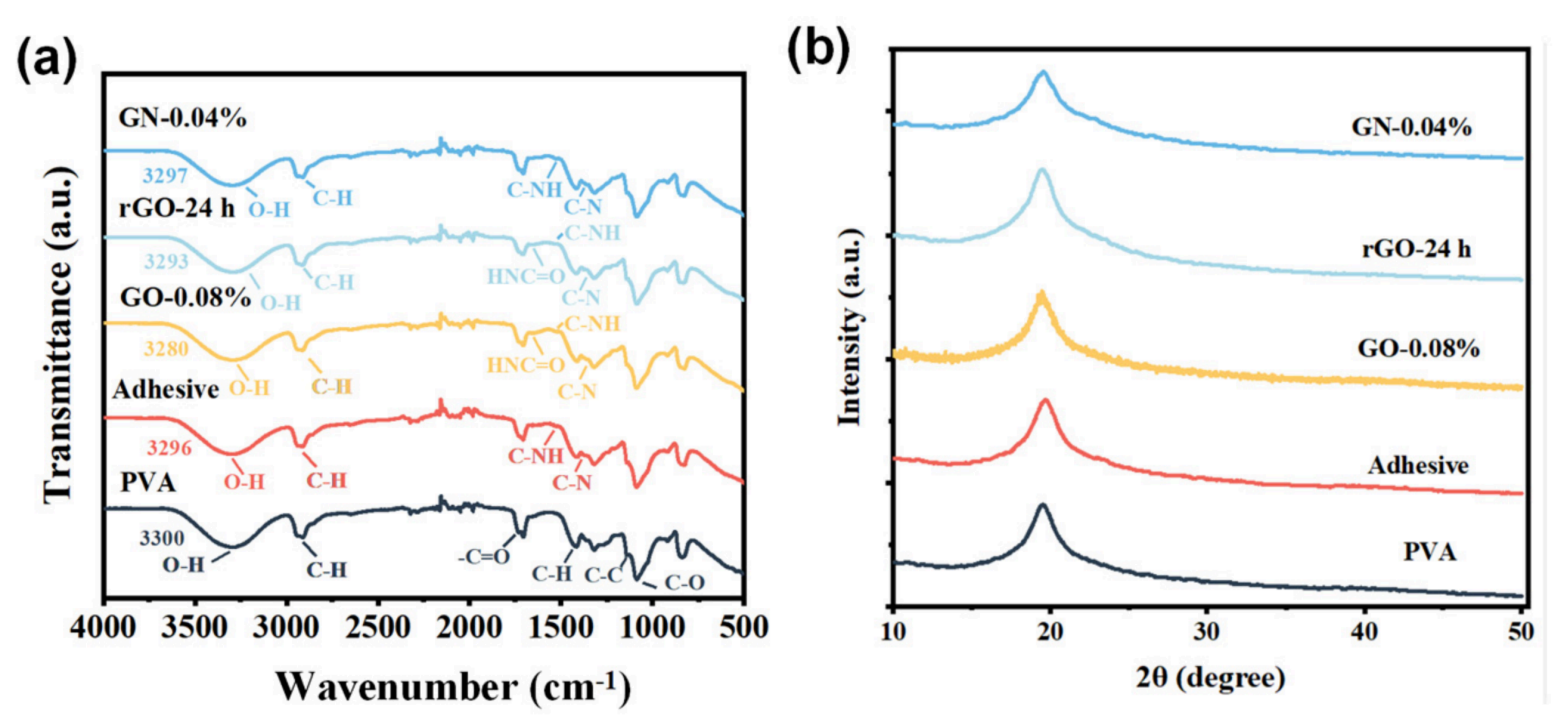
FT-IR (**a**) and XRD (**b**) spectra of PVA, adhesive, GO-0.08% adhesive films, rGO-24 h adhesive films, and GN-0.04% adhesive films.

As shown in Figure 3a, pristine PVA exhibits a broad O–H stretching band centered at approximately 3300 cm^−1^. After introducing PEI and ZrO(NO_3_)_2_, new absorption bands appear near 1640 cm^−1^ and 1550 cm^−1^, corresponding to amide-related HNC=O and C–NH vibrations, indicating successful crosslinking within the adhesive network. Upon incorporation of graphene derivatives, the O–H stretching peak gradually shifts from 3300 cm^−1^ (PVA) to 3296 cm^−1^ (adhesive), 3280 cm^−1^ (GO), 3293 cm^−1^ (rGO-AA), and 3297 cm^−1^ (GN). The pronounced red shift observed for GO indicates stronger hydrogen-bonding interactions, owing to its abundant oxygen-containing groups. In contrast, rGO-AA and GN exhibit smaller O-H shifts, suggesting reduced detectable hydrogen-bonding interactions associated with their lower oxygen contents.

It should be noted that the negligible O-H shift in GN compared with the blank adhesive does not indicate the complete absence of interfacial interactions. The O–H band displacement mainly reflects polar interactions involving oxygen-containing functionalities, whereas non-covalent interactions such as π–π interactions, van der Waals forces, and physical confinement effects are difficult to directly identify by FT-IR analysis. Therefore, the GN mainly contributes to the adhesive performance through its highly graphitized structure and intrinsic lamellar barrier effect rather than strong hydrogen-bond-mediated interactions. Similar oxygen-content-dependent interfacial interactions between GO and polymer matrices have been widely reported in graphene/polymer nanocomposites [27].

The XRD patterns shown in Figure 3b exhibit the characteristic diffraction peak of PVA at approximately 2θ = 20°. No significant shift in the diffraction peak position is observed after the incorporation of GO, rGO, or GN, indicating that the fundamental crystalline structure of the PVA matrix is maintained. Meanwhile, the calculated crystallinity index values (Table S2) exhibit only minor variations among PVA, adhesive, and graphene-derivative-modified adhesive films, suggesting that graphene incorporation induces only limited changes in the overall crystallinity of the PVA matrix. Therefore, although subtle variations in crystallinity may contribute to the moisture-barrier performance to some extent, the enhanced moisture resistance is primarily attributed to oxygen-content-dependent interfacial interactions and graphene-induced tortuous diffusion pathways.

Collectively, FT-IR and XRD analyses confirm that decreasing graphene oxygen content gradually weakens interfacial chemical interactions while enhancing graphitic barrier characteristics. GO primarily strengthens the adhesive network through hydrogen bonding and chemical coupling, whereas the GN mainly contributes through its intrinsic lamellar barrier effect, with rGO representing an intermediate state between the two.

### 3.2. Morphology and Dispersion Behavior of Graphene Derivatives

**Figure 4:**
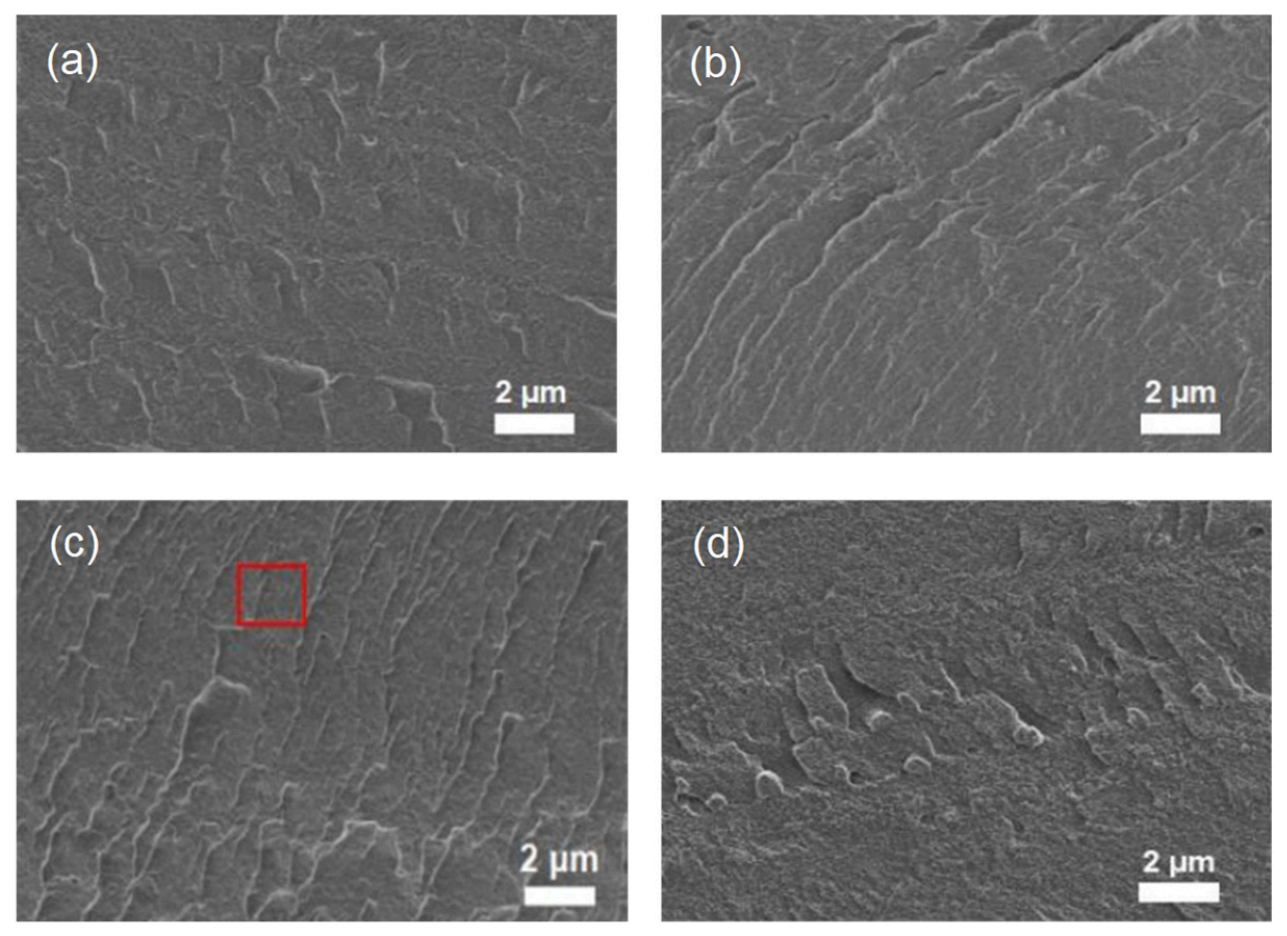
Cross-sectional FE-SEM images of (**a**) pure adhesive, (**b**) GO-0.08 wt% adhesive, (**c**) rGO-24 h adhesive, and (**d**) GN-0.04 wt% adhesive films. The red box in panel (**c**) highlights a typical region showing the dispersed rGO sheets within the matrix. Scale bars (2 μm).

To investigate the effect of graphene derivatives on the cross-sectional morphology and dispersion state of the adhesive matrix, FE-SEM characterization was performed. The representative cross-sectional images of the pure adhesive, GO-0.08 wt%, rGO-24 h, and GN-0.04 wt% adhesives are shown in Figure 4, while the full series of FE-SEM images for different GO concentrations, rGO reduction times, and GN loadings are provided in Figure S5 for completeness. During the preparation process, all graphene-modified adhesive formulations exhibited good dispersion stability without visible sedimentation or macroscopic aggregation. Moreover, the relatively low graphene loading enabled the maintenance of good processability and film-forming capability of the adhesive formulations. Prior to incorporation into the adhesive matrix, the morphology of graphene derivatives was examined by TEM (Figure S6). All graphene derivatives exhibited thin sheet-like structures without obvious large aggregation, confirming the preservation of their nanosheet morphology during the dispersion process.

The pure adhesive exhibits a relatively smooth cross-section with some minor cracks and defects. After incorporating GO, the cross-section becomes more compact with oriented lamellar structures, suggesting that GO nanosheets are well dispersed and aligned in the polymer matrix. This morphology is consistent with previous reports showing that GO nanosheets can establish strong hydrogen-bonding interactions with PVA chains, thereby promoting homogeneous dispersion and the formation of compact layered structures within the polymer matrix [28,29]. The rGO-24 h adhesive shows a similar layered morphology, with a uniform distribution of rGO sheets and no obvious aggregation. In contrast, the GN-0.04 wt% adhesive displays a denser and more homogeneous fracture surface, indicating that the highly graphitized GNs are evenly dispersed and effectively form a physical barrier network within the matrix. Similar lamellar architectures have been widely reported to increase the tortuosity of diffusion pathways and suppress moisture transport through polymer films. These morphological features are consistent with the trend observed across all tested concentrations and reduction times (Figure S5), suggesting that the dispersion behavior and lamellar structures are closely associated with the surface chemistry and structural characteristics of the graphene fillers.

### 3.3. Water Vapor Barrier Performance and Diffusion Regulation

**Figure 5:**
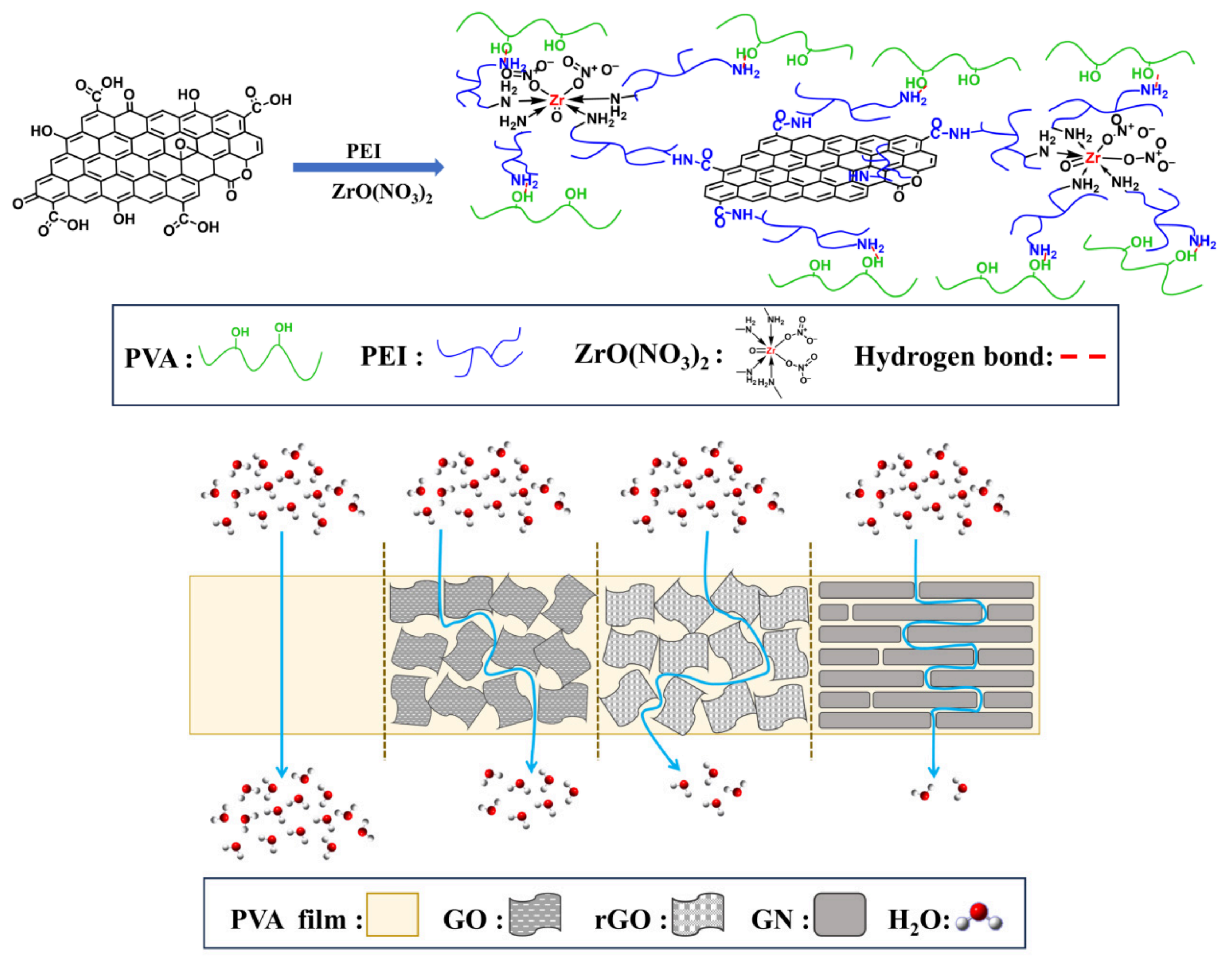
Schematic illustration of the proposed interfacial interaction network involving hydrogen bonding, polymer–graphene interactions, and possible Zr-mediated coordination interactions among PVA, PEI, ZrO(NO_3_)_2_, and graphene fillers (**top**), and the corresponding water vapor diffusion pathways in PVA, GO/PVA, rGO/PVA, and GN/PVA films (**bottom**). Symbols and legends are defined in the embedded boxes within the figure.

PVA inherently contains abundant hydrophilic hydroxyl groups, resulting in high water vapor permeability and poor moisture resistance, owing to the strong affinity of hydroxyl groups toward water molecules and the consequent facilitation of moisture sorption and diffusion [30]. To regulate moisture diffusion and improve environmental durability, graphene derivatives with progressively decreased oxygen contents (GO-0.08%, rGO-24 h, and GN-0.04%) were incorporated into the PVA/PEI/ZrO(NO_3_)_2_ adhesive system. The proposed interfacial interaction and water-barrier mechanisms are illustrated in Figure 5. The upper part presents the possible hybrid network involving hydrogen bonding, polymer–graphene interactions, and Zr-mediated coordination interactions, while the lower panel compares the different water diffusion pathways in neat PVA and graphene-modified composite films.

**Figure 6:**
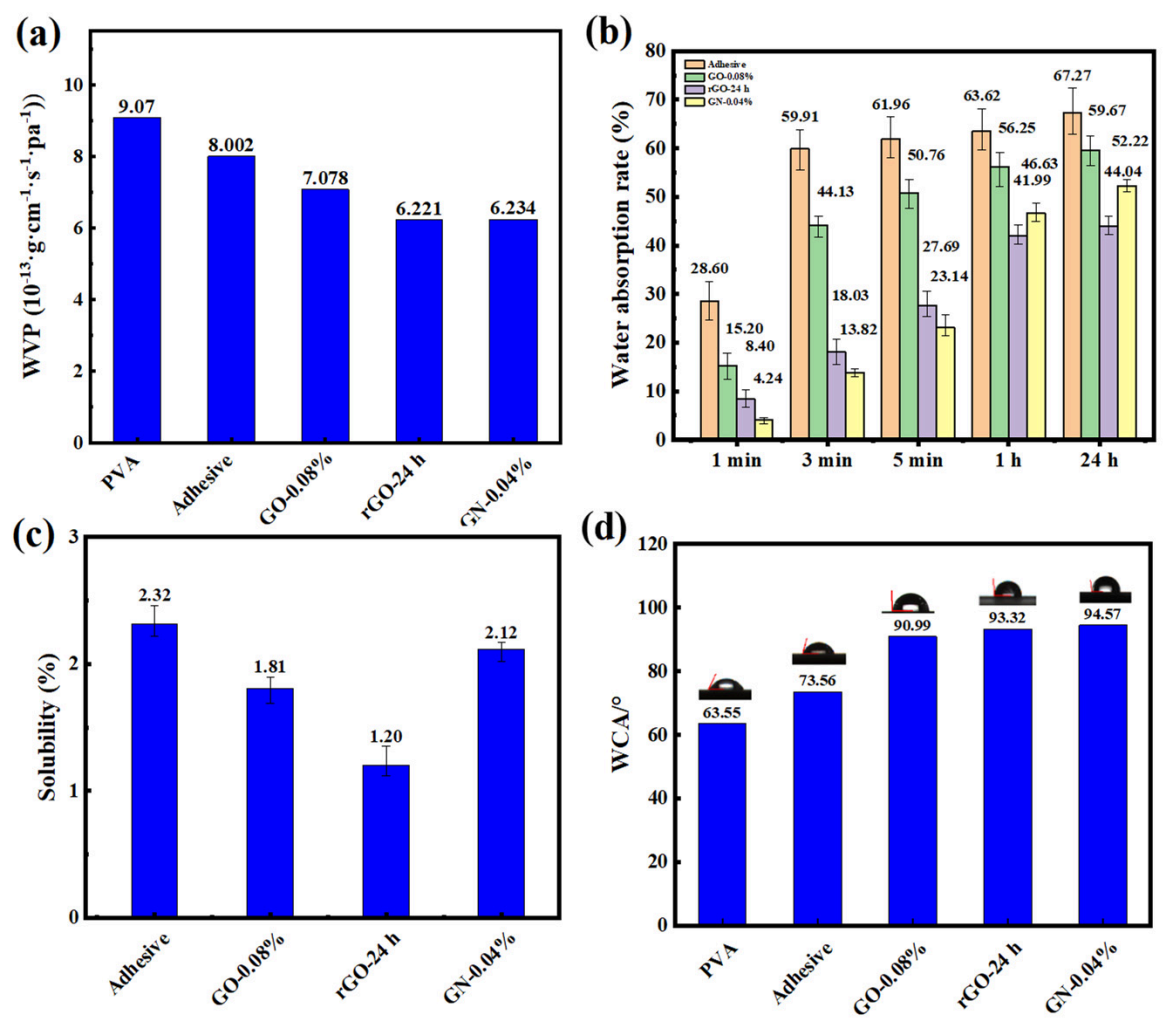
(**a**) Water vapor permeability (WVP), (**b**) water absorption rate, (**c**) water solubility, and (**d**) water contact angle (WCA) of the PVA-based adhesive films containing different graphene derivatives. Symbols and color legends for panel (**b**) are shown inside the figure. The small icons on top of bars in panel (**d**) are schematic illustrations of water-contact-angle measurement.

Systematic water vapor permeability (WVP) measurements with varied GO loadings, rGO reduction durations, and GN contents are summarized in Figure S7, while three representative samples (GO-0.08%, rGO-24 h, GN-0.04%) are presented here for comparison. As shown in Figure 6a, the crosslinked adhesive exhibits a lower WVP than pristine PVA, owing to the formation of a denser polymer network. Further incorporation of graphene derivatives significantly reduces water vapor permeability. Among them, rGO-AA exhibits the lowest WVP (6.221 × 10^−13^ g·cm·cm^−2^·s^−1^·Pa^−1^), followed by GN (6.234 × 10^−13^ g·cm·cm^−2^·s^−1^·Pa^−1^) and GO (7.078 × 10^−13^ g·cm·cm^−2^·s^−1^·Pa^−1^). The superior barrier performance of rGO-AA can be attributed to its balanced structure, in which residual oxygen-containing groups provide partial interfacial interactions with the PVA/PEI matrix, while the restored graphitic domains generate more tortuous diffusion pathways for water molecules [31]. Nevertheless, the reduction-induced loss of oxygen functionalities may also decrease the density of hydrogen-bonding interactions compared with GO, which could influence the long-term interfacial stability in practical polarizer assemblies.

Water absorption and solubility measurements further support this interpretation (Figure 6b,c). All graphene-modified adhesives exhibit lower water uptake and solubility than the pristine adhesive. Notably, rGO-AA shows the lowest water absorption and solubility (1.20%), indicating a more compact bulk adhesive network with reduced water uptake and restricted moisture penetration. However, these results mainly reflect the intrinsic moisture resistance of the adhesive layer and do not necessarily represent the long-term durability of the assembled polarizer structure, which is also strongly dependent on interfacial adhesion and multilayer structural stability. Although the GN possesses highly graphitized domains, its relatively weaker interfacial interactions with the polymer matrix led to slightly higher water absorption and solubility than rGO.

The surface wettability results shown in Figure 6d reveal a clear dependence on graphene oxygen content. The water contact angle increases from 90.99° for GO to 93.32° for rGO-AA and 94.57° for GN, demonstrating that the gradual removal of oxygen-containing groups effectively suppresses hydrophilic adsorption sites. Similar increases in surface hydrophobicity with decreasing oxygen content have been reported for graphene-based materials due to the progressive elimination of polar oxygen functionalities. Therefore, the GN exhibits the highest hydrophobicity among all samples.

### 3.4. Optical Transmittance and Antistatic Performance

**Figure 7:**
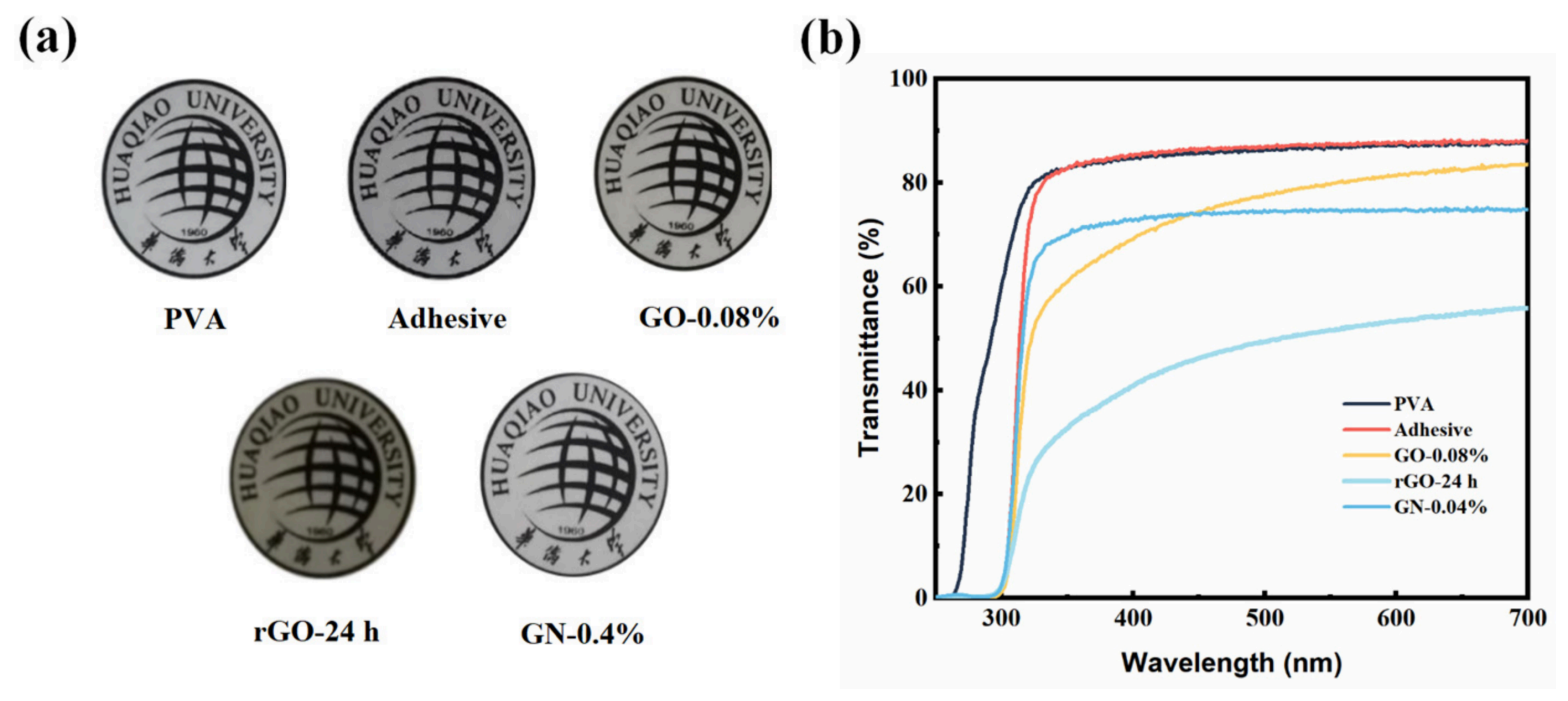
(**a**) Digital photographs of PVA, blank adhesive, GO-0.08%, rGO-24 h, and GN-0.04% adhesive films. The emblem stamp overlaid on each sample photograph is the official seal of Huaqiao University, which contains Chinese characters for institutional identification only. (**b**) UV–Vis transmittance spectra of the films in the wavelength range of 250–700 nm.

To evaluate the suitability of the graphene-modified adhesives for polarizer applications, their optical transmittance and antistatic properties were investigated. Based on the moisture-resistance results, GO-0.08%, rGO-24 h, and GN-0.04% were selected as representative samples for further evaluation. The digital photographs shown in Figure 7a demonstrate that all films maintain good transparency, allowing the underlying “Huaqiao University” logo to remain clearly visible. Compared with pristine PVA and the blank adhesive, the incorporation of graphene derivatives results in a certain degree of reduced transparency. Among the investigated samples, GO-0.08% retains the highest visible transparency, whereas rGO exhibits the most pronounced decrease in light transmission. GN maintains moderate transparency while preserving good film uniformity.

The UV–Vis transmittance spectra in Figure 7b further confirm these observations. Pristine PVA and the blank adhesive exhibit high transmittance throughout the visible region, whereas all graphene-modified films show reduced optical transmission. At 700 nm, GO-0.08%, GN-0.04%, and rGO-24 h exhibit visible transmittances of approximately 84%, 75%, and 56%, respectively. The significant transmittance reduction observed for rGO can be attributed to the partial restoration of π-conjugated graphitic domains after reduction, which enhances light absorption, together with increased light scattering from graphene nanosheets. In contrast, the GN maintains relatively high transparency despite its highly graphitized structure, likely owing to its lower loading level and uniform dispersion within the adhesive matrix. These results indicate that the optical properties are governed by both the graphitic structure and dispersion state of the graphene derivatives within the adhesive matrix.

The charge dissipation capability of the films was further evaluated by measuring their surface resistivity (Table 1). The surface resistivity was measured using a surface resistance tester with a two-electrode configuration, and the values were reported in Ω/sq. The detailed surface resistivity values of adhesive films with different graphene derivative contents and rGO reduction times are summarized in Table S3. Pristine PVA film exhibits a high surface resistivity of approximately 10^12^ Ω/sq, whereas the blank adhesive shows a slightly reduced value of approximately 10^11^ Ω/sq. This decrease can be attributed to the introduction of PEI and ZrO(NO_3_)_2_ into the adhesive network, which provide polar groups and ionic species that facilitate charge dissipation. After the incorporation of graphene derivatives, the surface resistivity further decreases due to the formation of graphitic charge-transfer pathways. GO-0.08% and rGO-24 h adhesive films exhibit surface resistivities of approximately 10^9^ Ω/sq, while GN-0.04% achieves the lowest value of approximately 10^8^ Ω/sq. The lower surface resistivity of the GN is associated with its highly graphitized structure, low oxygen content, and more continuous sp^2^ carbon domains, which facilitate electron transport within the adhesive matrix. According to commonly used surface resistivity classifications, all graphene-modified adhesive films exhibit static-dissipative behavior rather than conductive characteristics, suggesting their potential for reducing electrostatic accumulation in polarizer applications.

### 3.5. Application of Graphene-Modified Adhesives in Polarizer Base Films

Iodine-based polarizers are widely employed in display technologies, owing to their excellent optical performance; however, their long-term durability under humid conditions remains a major challenge. Moisture penetration through the adhesive layer can induce swelling, bubbling, delamination, and deterioration of the iodine–PVA complex, ultimately resulting in polarization failure. Therefore, the practical applicability of the graphene-modified adhesives was further evaluated using polarizer base films (POL-B) assembled with GO-0.08%, rGO-24 h, and GN-0.04% adhesives.

**Figure 8:**
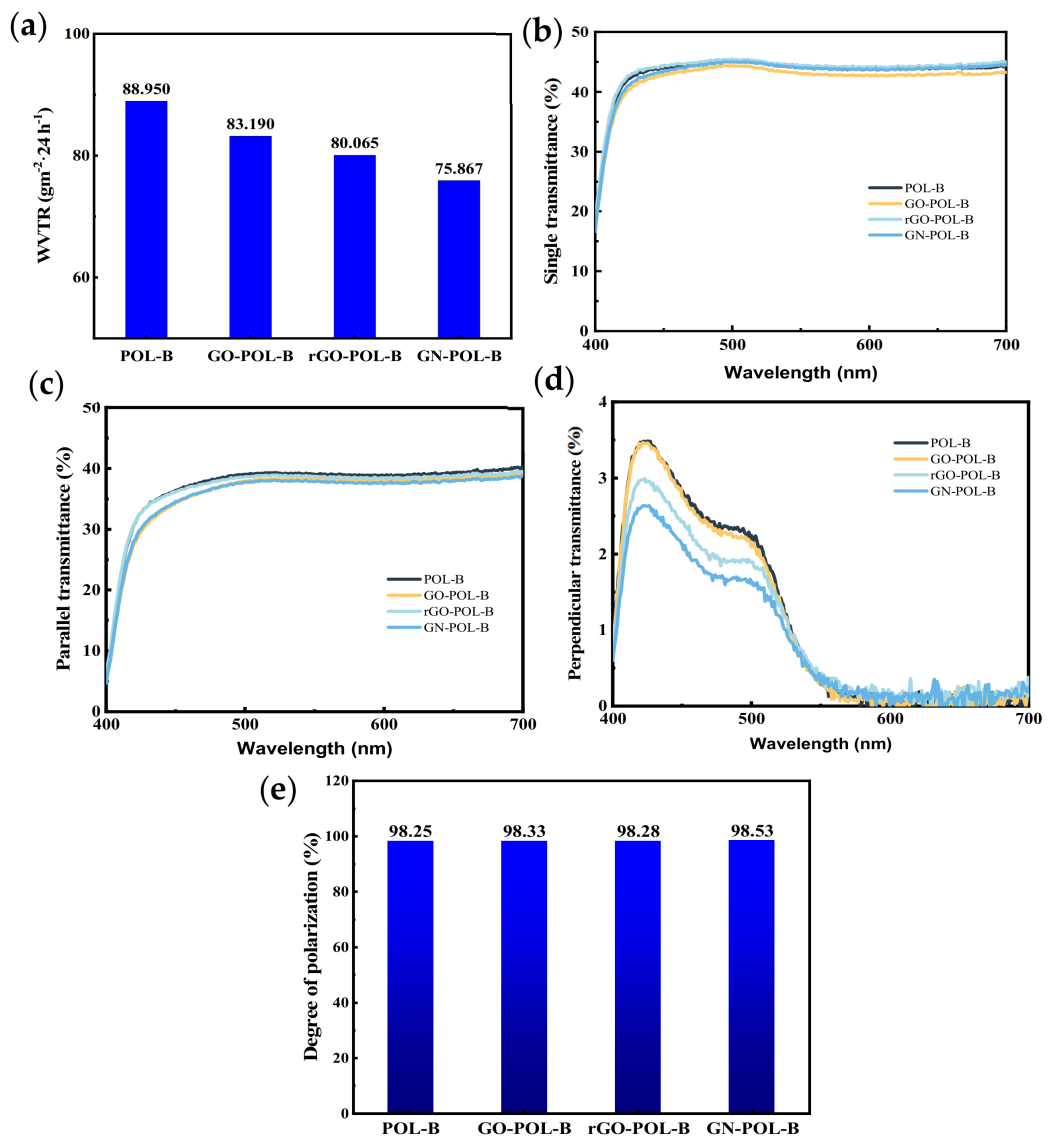
(**a**) Water vapor transmission rate (WVTR) of pristine and graphene-modified polarizer base films. (**b**) Single transmittance, (**c**) parallel transmittance, (**d**) perpendicular transmittance, and (**e**) polarization efficiency of the polarizer base films with different modified adhesives.

The water vapor transmission rate (WVTR) results of the polarizer base films are presented in Figure 8a. Compared with the pristine POL-B (88.950 g·m^−2^·24 h^−1^), all graphene-modified polarizers exhibit reduced moisture permeability. GO-POL-B and rGO-POL-B show moderate decreases to 83.190 and 80.065 g·m^−2^·24 h^−1^, respectively. Notably, GN-POL-B achieves the lowest WVTR of 75.867 g·m^−2^·24 h^−1^, corresponding to a 14.71% reduction relative to the pristine sample. This improved barrier performance is mainly attributed to the highly graphitized lamellar structure of the GN, which increases the tortuosity of moisture diffusion pathways. Moreover, the low oxygen content of the GN reduces water adsorption sites, thereby minimizing moisture accumulation within the adhesive layer during long-term exposure.

The optical properties of the modified polarizers were further characterized to verify that enhanced moisture resistance was achieved without compromising optical performance. As shown in Figure 8b–d, all graphene-modified polarizers exhibit comparable single transmittance (~43–45%), parallel transmittance (~38–40%), and low perpendicular transmittance relative to pristine POL-B, indicating that the introduction of graphene-modified adhesives does not adversely affect the optical characteristics of the polarizer films. The polarization efficiency (P) was further calculated according to Equation (S1) (SI) based on HG/T 4357-2012 [32]. As shown in Figure 8e and Table 2, all samples maintain high polarization efficiencies above 98%, including POL-B (98.25%), GO-POL-B (98.33%), rGO-POL-B (98.28%), and GN-POL-B (98.53%). These results confirm that the graphene-modified adhesives enhance moisture resistance while preserving the essential polarization capability of the polarizer base films. In addition, graphene-modified polarizers exhibit slightly reduced perpendicular transmittance, particularly in the short-wavelength region (Figure 8d), suggesting reduced optical leakage under crossed polarization conditions. Among the investigated samples, GN-POL-B shows the lowest perpendicular transmittance while maintaining comparable visible-light transmission, indicating a favorable balance between optical transparency and polarization performance.

To further evaluate the long-term environmental stability of the polarizer assemblies, a 40-day water-immersion test was conducted, as shown in Table S4. The pristine polarizer exhibited severe moisture-induced degradation, including obvious bubble formation, optical deterioration, and eventual interfacial delamination within 31 days. In comparison, all graphene-modified polarizer samples showed improved water resistance and structural stability. GO-POL-B developed visible bubbles after 6 days of immersion but maintained relatively intact interfacial adhesion throughout the test period, which may be related to the strong interfacial interactions and dense adhesive network induced by the oxygen-rich GO nanosheets. The rGO-POL-B sample remained stable during the initial immersion stage but gradually exhibited degradation after approximately 14 days. This behavior may result from the balance between moisture-barrier capability and interfacial compatibility. Although rGO effectively suppresses water diffusion through its restored graphitic domains, partial removal of oxygen-containing groups during the reduction process decreases the available sites for hydrogen-bonding interactions with the polymer matrix, which may influence the long-term stability of the adhesive–polarizer interface under prolonged humid exposure. In contrast, GN-POL-B showed only slight bubble formation after 8 days and maintained structural integrity without delamination throughout the entire 40-day immersion period, demonstrating the superior long-term moisture durability of the assembled polarizer structure. This enhanced durability is attributed to the synergistic contribution of the GN’s highly hydrophobic graphitic structure, reduced moisture adsorption, and stable lamellar barrier network, which effectively suppress moisture accumulation and maintain interface integrity during prolonged immersion.

It is noteworthy that although rGO-24h exhibits a slightly lower WVP in the isolated adhesive films, the GN delivers the best long-term performance in the assembled polarizer structures. This apparent discrepancy does not represent a contradiction, but rather highlights the different factors governing bulk adhesive properties and practical device durability. The moisture resistance of isolated adhesive films is primarily determined by diffusion resistance and water uptake behavior, whereas the long-term stability of polarizer assemblies additionally depends on interfacial adhesion, moisture accumulation, and the structural integrity of the TAC/adhesive/PVA multilayer system. For rGO-24 h, the restored graphitic domains effectively reduce water diffusion, resulting in excellent barrier performance; however, the reduced density of oxygen-containing groups may limit interfacial interactions under prolonged humid conditions. In contrast, the GN, with its highly graphitized structure, low oxygen content, and superior hydrophobicity, minimizes moisture adsorption and maintains a stable lamellar barrier network, leading to the best long-term protection of the polarizer structure.

## 4. Conclusions

In conclusion, graphene-based fillers with different oxygen contents and structural characteristics were incorporated into a PVA/PEI waterborne adhesive system to regulate interfacial interactions and moisture-barrier performance. The results demonstrate that the oxygen content of graphene fillers plays a critical role in balancing interfacial compatibility and barrier effects, thereby influencing the overall adhesive performance. Among them, the GN exhibited the most favorable combination of moisture resistance, antistatic capability, and optical preservation, benefiting from its highly graphitized structure and stable lamellar barrier effect. Furthermore, GN-modified polarizers demonstrated superior long-term immersion stability while maintaining excellent optical transmittance and polarization efficiency. This work provides insights into the design of graphene-modified waterborne adhesives for enhancing the environmental durability of iodine-based polarizers and other optoelectronic applications.

## Figures and Tables

**Table 1:** Surface resistivity of pure PVA film, blank adhesive film, and graphene-modified adhesive films (GO–0.08 wt%, rGO–24 h, and GN–0.04 wt%).

Sample	PVA Film	Adhesive Film	0.08% GO Adhesive Film	rGO-24 h Adhesive Film	0.04% GN Adhesive Film
Surface resistivity (Ω/sq)	1 × 10^12^	1 × 10^11^	1 × 10^9^	1 × 10^9^	1 × 10^8^

**Table 2:** Transmittance and polarization efficiency of pristine and graphene-modified polarizer base films.

Sample	Single Transmittance (%)	Parallel Transmittance (%)	Perpendicular Transmittance (%)	Polarization Efficiency (%)
POL-B	51.68	45.55	0.81	98.25
GO-POL-B	50.57	44.43	0.75	98.33
rGO-POL-B	52.11	45.09	0.78	98.28
GN-POL-B	51.63	44.03	0.65	98.53

## Data Availability

The original contributions presented in this study are included in the article/Supplementary Materials. Further inquiries can be directed to the corresponding authors.

## Supplementary Materials

The following supporting information can be downloaded at https://www.mdpi.com/article/10.3390/polym18151916/s1.
