# Supplementary material for: Oxygen-Content-Dependent Interfacial and Barrier Effects of Graphene Fillers in PVA Adhesives Toward Durable Polarizer Applications
Source: Polymers (Basel). 2026 Aug 5;18(15):1916. doi: 10.3390/polym18151916 (PMC13468561; doi:10.3390/polym18151916)
Supplement: Supplementary file 1 [file polymers-18-01916-s001.zip › polymers-4388482-supplementary.pdf]

Oxygen-Content-Dependent Interfacial and Barrier Effects of Graphene Fillers in PVA Adhesives toward Durable Polarizer Applications

Chang Sun[1†], Wentao Huang[1†], Ziyuan Zheng[1], Rui Huang,[2] Qinghua Zhao[1]*, Guohua Chen[1]*.

[1] College of Materials Science and Engineering, Huaqiao University, Xiamen 361021, China.

[2] Xiamen Xiangfuxing Technology Co., Ltd. Xiamen 361021, China.

* Correspondence: hdcgh@hqu.edu.cn or qhzhao@hqu.edu.cn

† Equally contributed to this work.

Supplementary Text

Calculation of transmittance and Polarization.

According to the standard HG/T 4357-2012 "Polarizer for Thin Film Transistor Liquid Crystal Displays (TFT-LCD)", and then combined with the bright visual function to calculate. Since the human eye can hardly perceive light in the wavelength band outside 700 nm to 400 nm, the wavelength interval of UV light used for the calculation is 700 nm to 400 nm. The spectra measured by UV-Vis were integrated in the range of 700 nm to 400 nm in combination with the correction of the human eye's visual function curve, and the optical average was calculated:

$$T = K\int_{400}^{700} P(\lambda)y(\lambda)T(\lambda)d\lambda \qquad (1)$$

$$K = \frac{100}{\int_{400}^{700} P(\lambda)y(\lambda)d\lambda} \qquad (2)$$

where P(λ) is the value of the spectral energy distribution of the standard light source, y(λ) is the isochromatic coefficient based on the XYZ system with a 2-degree field of view, and T(λ) is the value of the transmittance distribution.

Polarization efficiency (P) is a calculated value reflecting the integrated efficiency of polariser in producing polarised light, and is a very important functional parameter, which is calculated as follows:

$$P = \sqrt{\frac{T_{//} - T_{\perp}}{T_{//} + T_{\perp}}} \times 100\% \qquad \text{(Equation S1)}$$

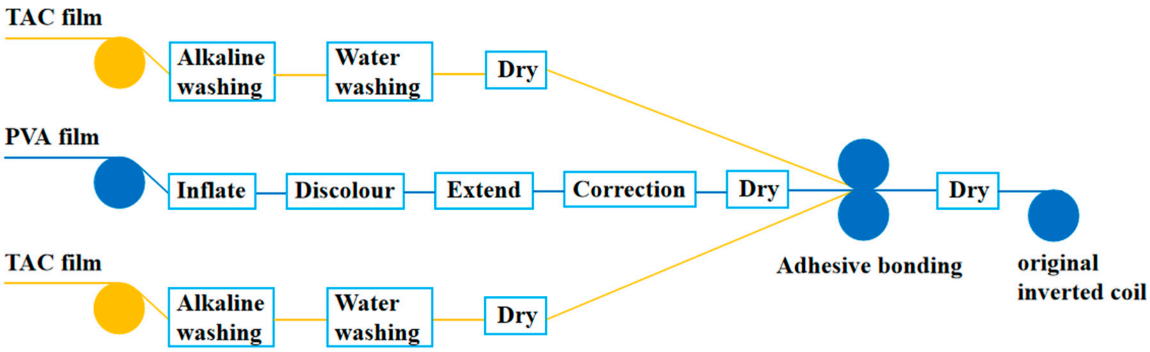

Figure S1. Polarized base film preparation process.

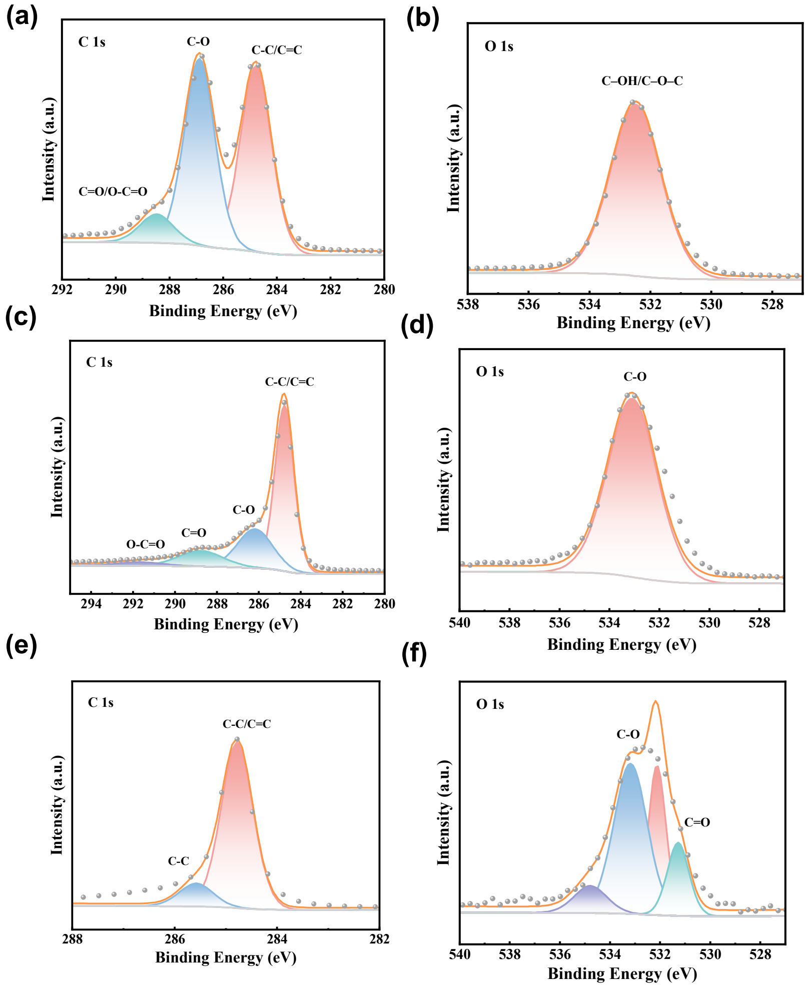

Figure S2. High-resolution XPS spectra of graphene derivatives. (a,c,e) C 1s spectra and (b,d,f) O 1s spectra of GO, rGO-24 h, and GN, respectively.

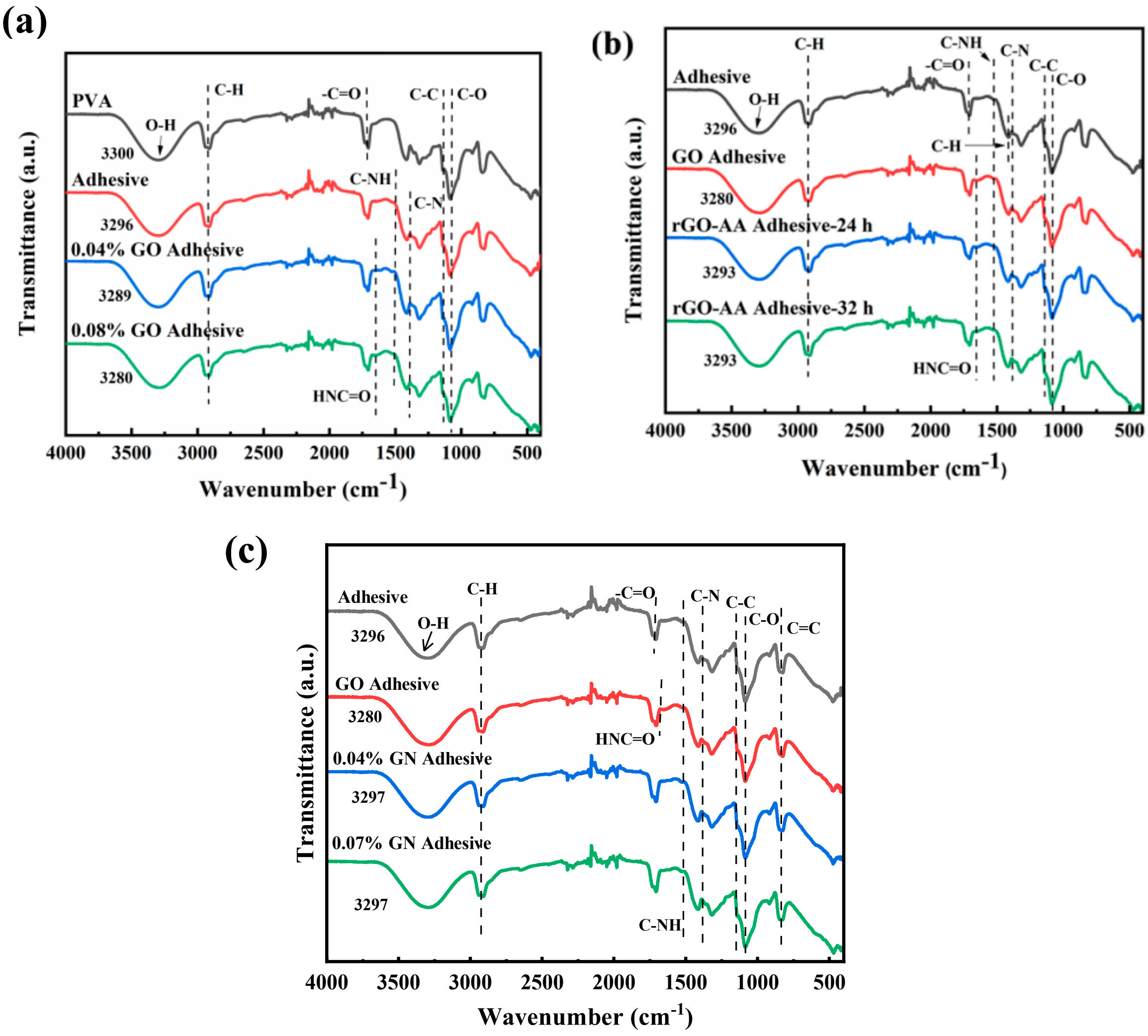

Figure S3. FT-IR spectra of GO-modified adhesive films with different GO concentrations (0, 0.04 wt%, and 0.08 wt%) (a), rGO-AA-modified adhesive films with different reduction times (0 h, 24 h, and 32 h) (b), and GN-modified adhesive films with different GN concentrations (0, 0.04 wt%, and 0.07 wt%) (c).

Supplementary text for Figure S3.

FT-IR spectra were used to analyze the structure of GO-modified adhesives (Fig. S3a). The characteristic peaks near 3300, 2923, 1730, 1141, and 1091 cm^{-1} correspond to the O–H, C–H, C=O, C–C, and C–O stretching vibrations of PVA, respectively. After crosslinking, the O–H stretching peak redshifts to 3296 cm^{-1}, indicating the formation of extensive hydrogen bonds. New peaks at 1530 cm^{-1} (C–NH) and 1376 cm^{-1} (C–N) appear, confirming the incorporation of PEI. Upon adding

GO, the characteristic signals of GO are covered by the polymer matrix, while the O–H peak further redshifts to 3289 cm^{-1}, and the C–NH peak at ~1530 cm^{-1} is slightly intensified. Meanwhile, a new shoulder peak at ~1648 cm^{-1}, assigned to the HNC=O stretching vibration, emerges. When the GO concentration increases to 0.08 wt%, the O–H peak continues to redshift to 3280 cm^{-1}, and the HNC=O peak at ~1648 cm^{-1} becomes significantly stronger. These results indicate that GO can form amide bonds with PEI and participate in hydrogen-bonding interactions within the adhesive, constructing a more compact crosslinked network.

The structure of rGO-AA-modified adhesives was characterized by FT-IR (Fig. S3b). The characteristic PVA peaks remain unchanged, while the rGO-AA adhesive obtained by crosslinking followed by 24 h reduction shows almost no change in the HNC=O (~1648 cm^{-1}) and C–N (~1376 cm^{-1}) stretching vibrations. However, the C–NH peak at ~1530 cm^{-1} weakens, while the C–N peak at ~1376 cm^{-1} is enhanced, indicating that PEI can undergo amide reactions normally during this process. When the reduction time is extended to 32 h, the intensities of the HNC=O and C–N peaks decrease, suggesting that prolonged reduction may affect the formation of amide bonds. Meanwhile, the O–H stretching peak of the GO adhesive (3280 cm^{-1}) slightly blueshifts to 3293 cm^{-1} with increasing reduction time, indicating that this reduction method has little disruption to the hydrogen-bonding network. Overall, the rGO-AA adhesive prepared by crosslinking followed by reduction maintains a favorable crosslinked structure.

FT-IR spectra of GN-modified adhesives are shown in Fig. S3c. The characteristic peaks of PVA remain unchanged after GN incorporation. The O–H stretching peak of both 0.04 wt% and 0.07 wt% GN adhesives is located at approximately 3297 cm^{-1}, showing negligible variation compared with the blank adhesive (3296 cm^{-1}). This result suggests limited hydrogen-bonding interactions between GN and the polymer matrix, consistent with the low oxygen content of GN. GN mainly contributes through its intrinsic lamellar barrier effect.

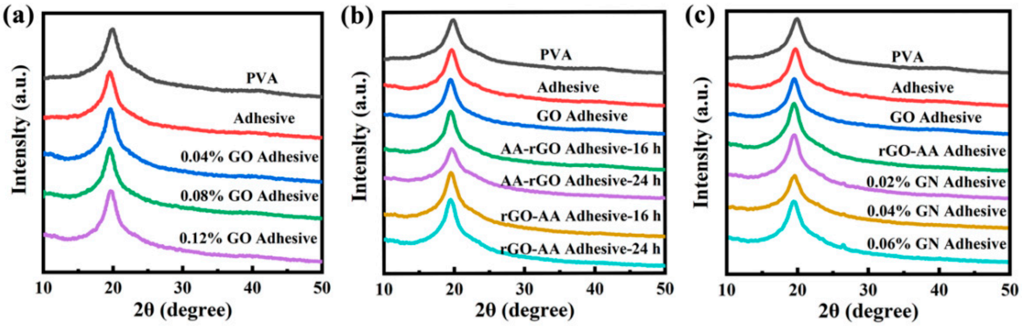

Figure S4. XRD spectra of (a) GO adhesive films, (b) rGO-AA adhesive films, and (c) GN adhesive films.

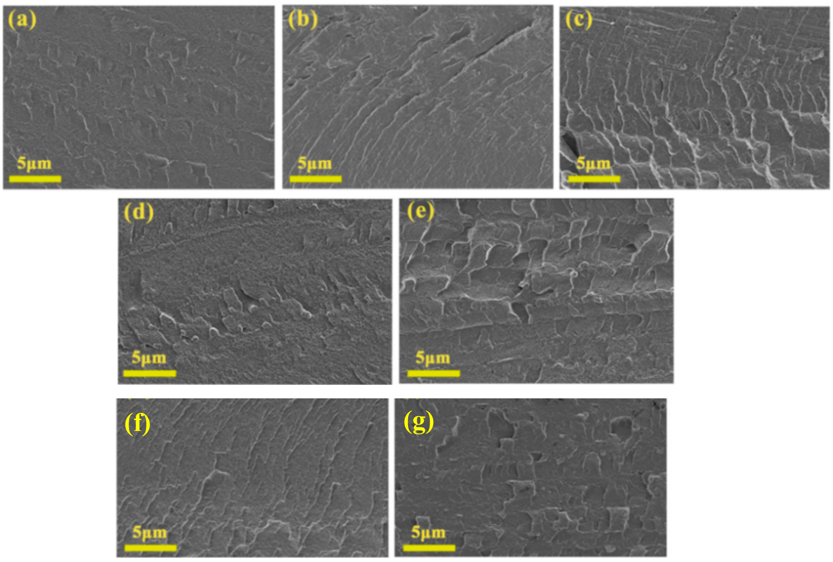

Figure S5. FE-SEM images of three adhesive film sections. (a) 0% GO adhesive film, (b) 0.08% GO adhesive film, (c) 0.12% GO adhesive film, (d) 0.04% GN adhesive film, (e) 0.06% GN adhesive film, (f) 24h rGO-AA adhesive film, (g) 32h rGO-AA adhesive films.

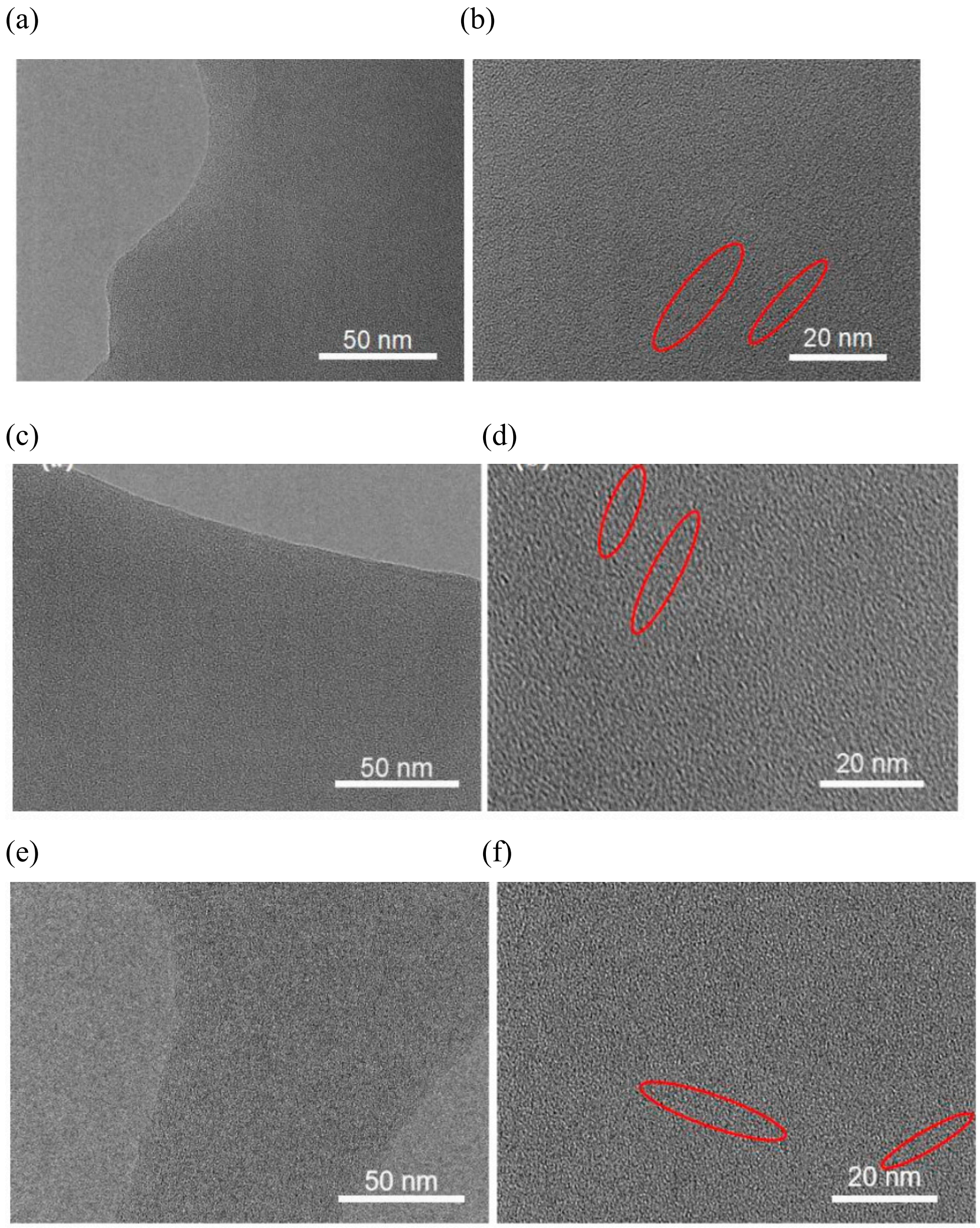

Figure S6. TEM images of graphene derivatives. (a,b) GO, (c,d) rGO, (e,f) GN.

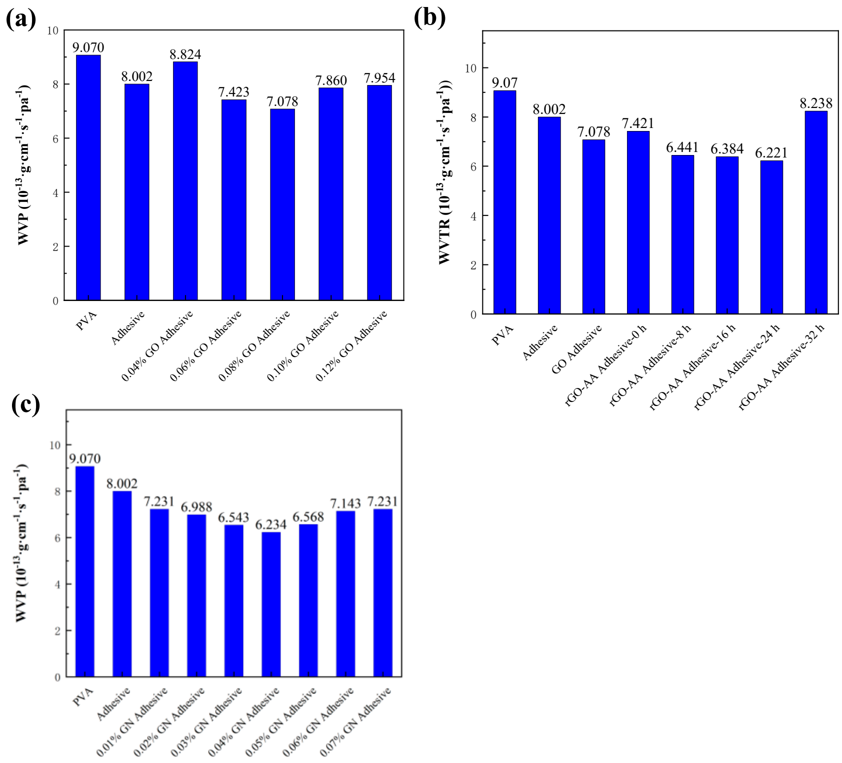

Figure S7. Water vapour permeability of three adhesive films. (a) GO adhesive films with different GO concentrations, (b) rGO-AA adhesive films with different reduction times, and (d) GN adhesive films with different GN concentrations.

<u>Supplementary text for Figure S7.</u>

Water vapor permeability (WVP) of GO-, rGO-AA-, and GN-modified adhesives with varying filler concentrations and reduction times is shown in Figure S6. For GO-modified adhesives (Figure S6a), WVP decreases gradually with increasing GO content up to 0.08 wt%, indicating that moderate GO loading enhances the crosslinked network and barrier performance. However, further increasing the GO concentration leads to slight aggregation and increased WVP. For rGO-AA adhesives (Figure S6b), the reduction time significantly affects the barrier performance, the 24 h reduction sample exhibits the lowest WVP, while excessive reduction weakens interfacial bonding and increases permeability. For GN-modified adhesives (Figure S6c), WVP decreases with increasing GN content up to 0.04 wt%, and then increases slightly at higher concentrations due to poor dispersion. These results confirm that the optimal barrier performance is achieved at specific GO concentration, reduction time, and GN loading, as selected in the main text.

Table S1. XPS-derived elemental composition and C/O atomic ratios of graphene derivatives.

Sample	C content (at%)	O content (at%)	C/O atmoc ratio
GO	59.31	40.69	1.46
rGO-24h	88.02	11.98	7.35
G2 (GN)	98.78	1.22	81.0

Table S2. Calculated crystallinity index values of PVA and graphene derivative-modified adhesive films.

Sample	Crystallinity index (%)
PVA	25.81
Adhesive	26.26
GO-0.08% adhesive	28.91
rGO-24 h adhesive	24.37
GN-0.04% adhesive	24.22

Calculation of crystallinity index:

The crystallinity index of the PVA-based adhesive films was calculated from the XRD patterns according to the following equation:

$$CI(\%) = \frac{A_c}{A_c + A_a} \times 100$$

The XRD patterns were analyzed in the range of 2θ=10-40°. After baseline correction, the crystalline contribution was determined by integrating the diffraction peak associated with the PVA crystalline phase around 2θ≈20°, while the remaining diffuse scattering area was assigned to the amorphous phase. The calculated crystallinity index values of PVA, blank adhesive, and graphene derivative-modified adhesive films are summarized in Table S2.

Furthermore, since the samples are multicomponent adhesive systems containing PVA, PEI, $ZrO(NO_3)_2$, and graphene derivatives, the obtained values are reported as calculated crystallinity indices rather than absolute crystallinity values.

Table S3. Surface resistivity of adhesive films with different graphene derivatives.

Sample	Surface resistivity (Ω/sq)	Sample	Surface resistivity (Ω/sq)	Sample	Surface resistivity (Ω/sq)
PVA film	1×10^{12}	0h rGO-AA adhesive film	1×10^{9}	0.01% GN Adhesive film	1×10^{9}
Adhesive film	1×10^{11}	8h rGO-AA adhesive film	1×10^{9}	0.02% GN Adhesive film	1×10^{9}
0.04% GO Adhesive film	1×10^{10}	16h rGO-AA adhesive film	1×10^{8}	0.03% GN Adhesive film	1×10^{8}
0.06% GO Adhesive film	1×10^{9}	24h rGO-AA adhesive film	1×10^{8}	0.04% GN Adhesive film	1×10^{8}
0.08% GO Adhesive film	1×10^{9}	32h rGO-AA adhesive film	1×10^{8}	0.05% GN Adhesive film	1×10^{8}
0.10% GO Adhesive film	1×10^{9}	/	/	0.06% GN Adhesive film	1×10^{8}
0.12% GO Adhesive film	1×10^{9}	/	/	0.07% GN Adhesive film	1×10^{8}

Supplementary text for Table S3

The addition of GO effectively reduces the surface resistance of the adhesive film, and as the GO concentration is increased from 0.04% to 0.06%, the surface specific resistance of the GO adhesive film decreases by two orders of magnitude compared to the adhesive film. The surface specific resistance of the two rGO adhesive films (reduced for 16 h) reached 10^{8} Ω/sq, which was 4, 3 and 1 orders of magnitude lower compared to the PVA, adhesive and GO adhesive films, respectively. The surface specific resistance decreased by 2 orders of magnitude after the addition of GN compared to the adhesive film, and the surface specific resistance continued to decrease by 1 order of magnitude after the GN concentration was increased to 0.03%, but did not continue to decrease as the GN concentration continued to increase.

Table S4. Water resistance test for modified polarizer base film

Soaking time/d	POL-B	GO-POL-B	rGO-POL-B	GN-POL-B
0	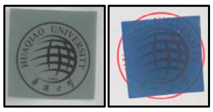	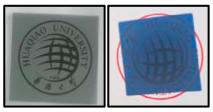	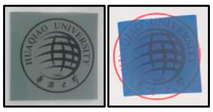	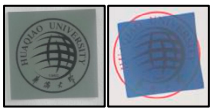
2	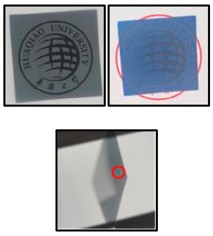	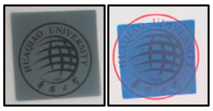	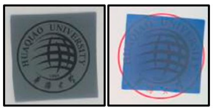	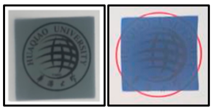
6	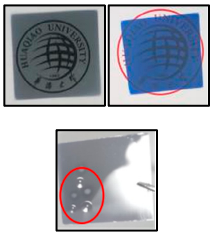	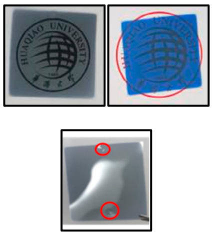	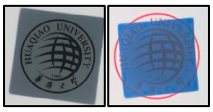	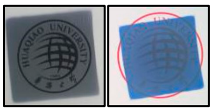
7	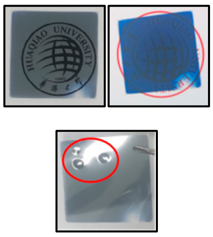	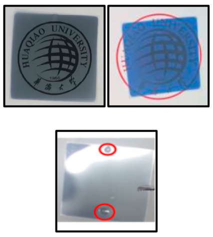	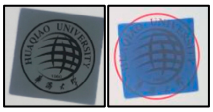	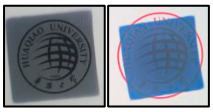
8	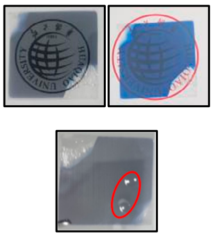	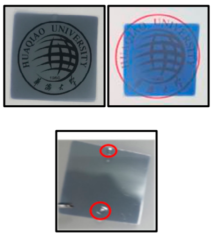	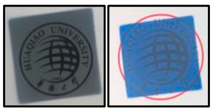	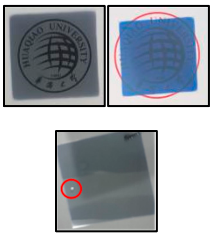
14	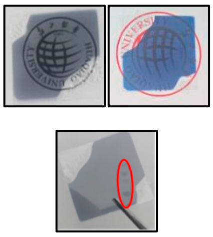	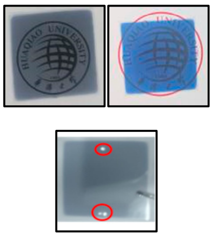	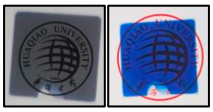	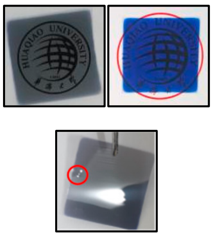
25	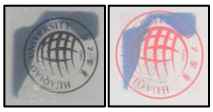	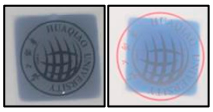	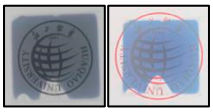	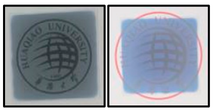

	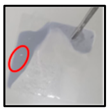	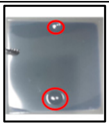	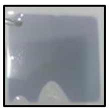	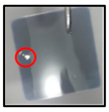
**31**	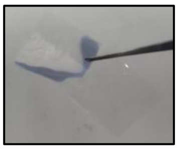	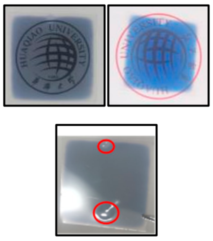	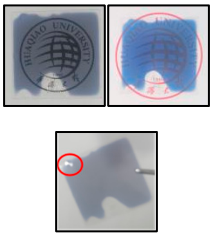	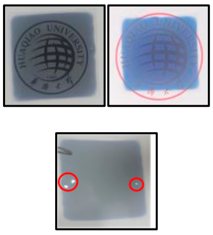
**40**	/	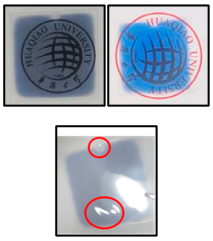	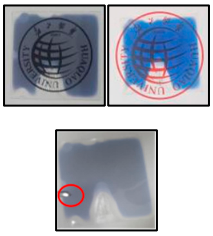	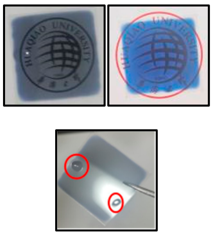

<u>Supplementary text for Table S4</u>

The diagrams in the table show, from left to right, from top to bottom, the specimen placed on A4 paper with the school logo printed on it, placed on a tablet PC with the angle adjusted to the darkest point (the light-absorbing axis of the polarised base film is perpendicular to the light-absorbing axis of the polarising structure in the tablet PC structure), and clamped on tweezers and photographed to observe defects in the specimen. The polarised base film started to bubble after 2 d of immersion, and the bubbles gradually increased as the immersion time became longer and longer. After 7 d of immersion, the polarised base film was biased. When the immersion time was extended to 31 d, the polarised base film became more biased, and the phenomenon of debonding occurred, and the film structure was separated. The GO polarised base film started to bubble after 6d of immersion, while the GN polarised base film started to bubble after 8d of immersion, but both polarised base films remained free from bias fading and debonding after 40 d of immersion. However，the

rGO polarising substrate film experienced fading bias after 14 d of immersion, and after 40 d of immersion, the rGO polarising substrate film experienced debonding.
